# Neural correlates of lexical stress processing in a foreign free‐stress language

**DOI:** 10.1002/brb3.2854

**Published:** 2022-12-26

**Authors:** Sandra Schwab, Michael Mouthon, Lea B. Jost, Justine Salvadori, Ilona Stefanos‐Yakoub, Eugénia Ferreira da Silva, Nathalie Giroud, Benoit Perriard, Jean‐Marie Annoni

**Affiliations:** ^1^ Department of French University of Fribourg Fribourg Switzerland; ^2^ Neurology‐Laboratory for Cognitive and Neurological Sciences University of Fribourg Fribourg Switzerland; ^3^ Computational Neuroscience of Speech & Hearing, Department of Computational Linguistics University of Zurich Zürich Switzerland

**Keywords:** default mode network, fMRI, foreign/second language acquisition, frontotemporal areas, lexical stress processing, speech processing, stress “deafness”

## Abstract

**Introduction:**

The paper examines the discrimination of lexical stress contrasts in a foreign language from a neural perspective. The aim of the study was to identify the areas associated with word stress processing (in comparison with vowel processing), when listeners of a fixed‐stress language have to process stress in a foreign free‐stress language.

**Methods:**

We asked French‐speaking participants to process stress and vowel contrasts in Spanish, a foreign language that the participants did not know. Participants performed a discrimination task on Spanish word pairs differing either with respect to word stress (penultimate or final stressed word) or with respect to the final vowel while functional magnetic resonance imaging data was acquired.

**Results:**

Behavioral results showed lower accuracy and longer reaction times for discriminating stress contrasts than vowel contrasts. The contrast Stress > Vowel revealed an increased bilateral activation of regions shown to be associated with stress processing (i.e., supplementary motor area, insula, middle/superior temporal gyrus), as well as a stronger involvement of areas related to more domain‐general cognitive control functions (i.e., bilateral inferior frontal gyrus). The contrast Vowel > Stress showed an increased activation in regions typically associated with the default mode network (known for decreasing its activity during attentionally more demanding tasks).

**Conclusion:**

When processing Spanish stress contrasts as compared to processing vowel contrasts, native listeners of French activated to a higher degree anterior networks including regions related to cognitive control. They also show a decrease in regions related to the default mode network. These findings, together with the behavioral results, reflect the higher cognitive demand, and therefore, the larger difficulties, for French‐speaking listeners during stress processing as compared to vowel processing.

## Graphical abstract

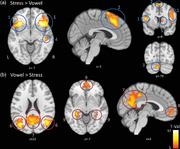

## 1 INTRODUCTION

The discrimination of lexical stress contrasts in a foreign/second language (L2) (e.g., en. *import* vs. en. *import*; es. *válido* vs. *valido*, en. valid versus I validate)^1^ constitutes a complicated task for some listeners, especially for native speakers of languages with predictable stress (e.g., French, Hungarian). This observation has led to the hypothesis than listeners of languages with predictable stress can be “deaf” to stress to some extent (Dupoux et al., 1997). Previous research has focused on investigating language‐, rhythm‐, or cognition‐related factors that may affect the listeners’ ability to detect stress in a foreign language (e.g., listeners’ native language, musical aptitude, working memory or phonological awareness; Degrave, 2017; Dupoux et al., 1997; Kolinsky et al., 2009; Honbolygó et al., 2019). In this paper, we examine the discrimination of L2 stress contrasts from a neural perspective. To our knowledge, the behavioral conclusions about stress “deafness” have not yet been investigated on the neural level. The present research is thus the first attempt to localize the brain areas involved in French‐speaking listeners’ stress processing.

Lexical stress (also called word stress) is the accentuation of a syllable within a word. For example, the word *prosody* has stress on the first syllable (i.e., *prosody*), whereas *prosodic* has stress on the second syllable (i.e., *prosodic*). Perceptually, the stressed syllable is more salient than the unstressed syllables of a word. Stress plays an important role in speech segmentation and in word recognition (Cutler, 2005), and hence, in speech comprehension in general.

The stress properties of French and Spanish—the two languages under study in this research—differ in several respects. Spanish is a free‐stress language (Quilis, 1993) where the position of stress within the words is determined by morphophonological constraints and varies from one word to another (e.g., es. *rosa*, en. rose, es. *correr*, en. run). For that reason, free‐stress languages like Spanish are said to show “variable” stress. As a consequence, stress can play a distinctive role in these languages, because the stressed syllable can be the only difference between two words (e.g., es. *válido* vs. *valido*, en. valid vs. I validate). In contrast, French is a fixed‐stress language (Lacheret‐Dujour & Beaugendre, 1999), where the position of stress does not vary from word to word. French stress is predictable, because it always falls on the final syllable of the word or group of words. Therefore, stress does not have a distinctive function. French differs, however, from other fixed‐stress languages (e.g., Hungarian, Polish) in that stress, in continuous speech, is not a word stress, but a “group stress”: It does not fall on each content word of the utterance, but at the end of the accentual group (e.g., *le joli petit chat dort sous la table*, en. the nice little cat sleeps under the table, where stresses fall on *chat* and *table*, which are at the end of accentual groups).

Native speakers of languages in which the position of stress is predictable (e.g., French, Hungarian, Korean) have been shown to have difficulties in identifying the position of lexical stress in a foreign language for which the position of stress is variable (e.g., English, German, Spanish) (Dupoux et al., 1997; Honbolygó et al., 2019; Schwab & Dellwo, 2017). The so‐called stress deafness that some listeners experience is due to the fact that, because stress is predictable in their native language, listeners have not developed strategies that allow them to encode stress information for lexical items (Dupoux et al., 1997; Schwab & Dellwo, 2017). In other words, because stress does not generate phonological distinctions in their native language, they do not use stress information in lexical processing. On the contrary, native speakers of English, Spanish, or German—languages with variable stress—have developed a mechanism for processing stress patterns. It is indeed well established that stress information influences how words are recognized in these languages. Several studies have shown that in languages with variable word‐level stress (e.g., English, German, Spanish), stress constrains lexical access (e.g., Cooper et al., 2002; Cutler & Pasveer, 2006; Cutler et al., 2007; Soto‐Faraco et al., 2001). The recognition of words that match the input both segmentally and suprasegmentally is facilitated as compared to the recognition of words that only match the segmental input (e.g., Cooper et al., 2002; Soto‐Faraco et al., 2001).

As a consequence, native speakers of languages with variable stress experience little difficulty in discriminating lexical stress contrasts in a foreign language (Dupoux et al., 1997). However, it has been shown that their performance in L2 does not reach the performance of native speakers, which has been accounted for by the native language (L1), L2 default stress patterns and the acoustic cues used to signal stress in L1 and L2. For example, Ortega‐Llebaria et al. (2013) and Schwab and Dellwo (2017) found that English and German listeners did not perform as well as native Spanish listeners in a Spanish stress detection task. The authors explained this poorer performance by the fact that the English and German languages have phonological vowel reduction (i.e., vowels are reduced in unstressed syllables), whereas Spanish does not. Therefore, native speakers of English and German could not use the information relative to vowel reduction to detect stress in Spanish the way they were used to do in their native language.

Neuroimaging studies supplement the conclusions drawn in behavioral studies. ERP studies have demonstrated that French advanced learners of German, contrary to native German listeners and Spanish learners of German, were not sensitive to metrical violations of German regular trochaic stress pattern (i.e., absence of P600) (Schmidt‐Kassow, Roncaglia‐Denissen, et al., 2011; Schmidt‐Kassow, Rothermich, et al., 2011). Moreover, the listeners’ stress detection abilities have been shown to be linked to working memory processing (i.e., P300), but not to auditory processing (i.e., N200) (Schwab et al., 2020). From such studies, it remains unclear, however, which neural networks are activated in L2 stress perception. In the present study, we used functional magnetic resonance imaging (fMRI), with better spatial resolution than EEG, to further investigate the neuronal networks implicated in L2 stress perception.

When it comes to stress detection, different processes seem to be involved for sentence versus word stress processing. Although it has been demonstrated that the processing of sentence melody activates auditory‐related areas particularly in the right hemisphere (e.g., Meyer et al., 2002), the neural correlates underlying word stress processing are less well understood. To date, word stress processing has primarily been associated not only with activations in the superior temporal gyrus (STG) and superior temporal sulcus (STS), but also with more frontal areas (inferior frontal, pre‐and postcentral, and middle frontal gyrus) (Aleman et al., 2005; Domahs et al., 2013; Heisterueber et al., 2014; Kandylaki et al., 2017; Klein et al., 2011; Honbolygó et al., 2020). Regarding the lateralization of word stress processing, it seems too early to draw strong conclusions given the diverse results. Several previous studies on healthy participants (e.g., Aleman et al., 2005; Klein, et al., 2011, Honbolygó et al., 2020) as well as clinical populations (Gandour & Baum, 2001; Häuser & Dohmas, 2014) point toward a lateralization of activation to the left hemisphere during stress processing. However, other studies have also found bilateral activation (e.g., Heisterueber et al., 2014), especially with increasing task difficulty (e.g., Domahs et al., 2013; Klein et al., 2011).

Of particular interest for the present research is the study by Klein et al. (2011), who compared the brain regions activated during stress and vowel processing in German L1. They used a discrimination task where German participants had to discriminate German pseudowords that differed in stress pattern (e.g., bokam‐bokam) or in vowel quality (e.g., bokam‐bekam). The authors observed a frontotemporal network basically comprising the right STG and the bilateral inferior frontal gyri to be specifically associated with stress processing. They suggested that there is a basic system for word stress processing in the left hemisphere, which tends to be supported by the right hemisphere in the case of increasing task difficulty.

The data in Klein et al. (2011) have been obtained with native listeners of a free‐stress language (i.e., German) who were familiar with stress allowing the distinction between words. As previously mentioned, native listeners of languages with predictable stress (e.g., French) are considered to be “deaf” to stress (e.g., Dupoux et al., 1997). In order to extend Klein and colleagues’ findings to foreign language processing, we investigated in the present study the neural correlates of stress processing in a foreign free‐stress language by French‐speaking listeners. More specifically, we asked French‐speaking participants to process stress and vowel contrasts in Spanish, a foreign language that the participants did not know. Contrary to Klein et al. (2011)’s study, the French‐speaking participants of the present experiment do not use stress information to distinguish words in their native language. However, like the German participants in Klein et al. (2011), native speakers of French use vowel quality to distinguish words (e.g., fr. *mort* vs. *mer*, en. death vs. sea). French listeners are therefore able to use vowel information, but not stress information to discriminate words in their native language. Consequently, according to stress “deafness” behavioral results, we assume that stress processing in Spanish will be more difficult for them than vowel processing. For that reason, we predict a bilateral activation of regions previously associated with stress processing (i.e., temporal areas, pre‐/postcentral cortex and insula; Klein et al., 2011), as well as a stronger involvement of areas related to more domain‐general cognitive control functions (e.g., Fedorenko & Blank, 2020).

## 2 METHOD

### 2.1 Participants

Thirty students participated in the experiment (18 women; range = 18–27 years; mean age = 22.67; st. dev. = 2.37). They were all recruited in Switzerland at the University of Fribourg or Haute Ecole in Fribourg via announcements on social media. Participants were native speakers of Swiss or standard French. They had no knowledge of Spanish, Italian, or Portuguese (i.e., free‐stress romance languages). Because German and English are mandatory disciplines in the Swiss educational system, all participants had school knowledge of these two languages. Given that musical expertise has been shown to influence stress processing (e.g., Degrave, 2017), we controlled for the participants’ musical aptitude. Although 13 participants had received musical training (among which 7 were still playing an instrument at the moment of the experiment), none of them was a professional musician. All participants were paid for their participation. The study received ethics approval from the Ethics Committee of the Psychology Department of the University of Fribourg.

### 2.2 Material

Twenty‐four Spanish lexemes (i.e., verbs) were used in three inflected verbal forms. The *1sg present indicative* forms (e.g., *camin*o, en. I walk) consisted of words with penultimate stress ending in the vowel *‐o* (i.e., “penult stressed o‐words”). The *3sg simple past indicative* forms (e.g., *caminó*, en. he/she walked) were words stressed on the final syllable ending with *‐o* (i.e., “final stressed o‐words”). The *1sg and 3sg present subjunctive* forms (e.g., *camine*, en. that I/he/she walk(s)) consisted of words with penultimate stress ending in the vowel *‐e* (i.e., “penult stressed e‐words”). Penult stressed o‐words and final stressed o‐words were thus segmentally similar but different regarding their stress pattern, whereas penult stressed o‐words and penult stressed e‐words were different segmentally, but shared the same stress pattern.

The 72 words (24 lexemes × 3 inflected forms) were recorded several times in isolation by a female native Speaker of Castilian Spanish. We selected the two best tokens for each penult stressed o‐word and each penult stressed e‐word, as well as the best token for each final stressed o‐word. We equalized the duration of all words to be 600 ms using Praat scripts (Boersma & Weenink, 2018).

Each trial was composed of a word pair (600 ms each word) separated by 500 ms, for a total duration of 1700 ms. We created word pairs that were divided into two conditions (i.e., stress and vowel). In both conditions, half of the word pairs were the same, and the other half were different. In the “stress” condition, the “different” word pairs differed only with respect to word stress. They were composed of a penult‐stressed o‐word (e.g., *camino*) and a final stressed o‐word (e.g., *caminó*). The “same” word pairs consisted of two different tokens of the same penult‐stressed o‐word (e.g., *camino*–*camino*). In the “vowel” condition, the “different” word pairs differed only with respect to the final vowel quality (i.e., stress pattern was the same in the two words). They were composed of a penult‐stressed o‐word (e.g., *camino*) and a penult‐stressed e‐word (e.g., *camine*). The “same” word pairs consisted of two different tokens of the same penult‐stressed e‐word (e.g., *camine*–*camine*).

The 96 word pairs (24 lexemes × 2 conditions × 2 same/different) were duplicated with the inverse presentation order within the pair, leading to a total of 192 word pairs. For each condition, we created 16 blocks of six word pairs stemming from different lexemes. Each block contained three same and three different word pairs. Blocks within each condition never contained the same six word pairs. Stress and vowel blocks alternated. Additionally, 12 practice word pairs were created, among which 6 were same and 6 were different.

Given the lexical overlap between Spanish and French, most of the Spanish lexemes were cognates, as they presented phonological and semantic similarity with French lexemes (e.g., es. liberar—fr. libérer). The effect of cognates has been shown to affect auditory L2 word recognition, especially in lexical decision tasks (Cornut et al., 2022; Muntendam et al., 2022). However, we assume the effect to be controlled in the present discrimination experiment by the fact that both pair members (e.g., *libero‐liberó* or *libero‐libere*) came from the same lexeme (i.e., verb; e.g., liberar) and, thus, were both cognates. Along the same lines, it is also well known that lexical frequency and phonological neighborhood density/frequency impact L2 word recognition, although to a different extent depending on learners’ L2 proficiency (Bradlow & Pisoni, 1999; Diependaele et al., 2013; Imai et al., 2005; Llompart, 2021). Because the participants of the present experiment had no knowledge of Spanish, we do not think that the influence of these factors biased their responses. Moreover, given that both words to be discriminated stemmed from the same lexeme (i.e., verb), and were also phonological neighbors, they shared the same lexical frequency as well as the same phonological neighborhood density/frequency.

### 2.3 Procedure

#### 2.3.1 Data collection

E‐Prime 3.0 (Psychology Tools, Inc., Pittsburgh, PA, USA) was used for controlling stimulus presentation and response recording. Participants performed a discrimination task. After hearing word pairs, they were asked to indicate whether the two words were the same or different by pressing a button with their right hand.

As previously mentioned, the experiment was composed of 32 blocks, with the alternation of Vowel and Stress blocks. Each block began with the display (during 1000 ms) of the information of the condition (Vowel or Stress) that would be presented in the block, followed by the presentation of a cross during 1000 ms. Then, the six 1700 ms trials were presented with a constant ISI of 2000 ms. The duration of a block was of 22.2 s followed by a blank screen of 8 s. The experiment lasted approximately 18 min divided into two equal scan runs with a 1‐min break in‐between. The order of presentation of the blocks within a run was randomized and alternated between experimental conditions. The order of runs was counterbalanced across participants. To get familiar with the task, participants were trained outside the scanner on 12 items that were not part of the stimulus material of the experiment.

#### 2.3.2 fMRI data acquisition

A 3T MRI scanner (Discovery MR750; GE Healthcare, Waukesha, Wisconsin) equipped with a 32‐channel head coil was used to acquire the data. The instructions were displayed on an LCD screen (32″ NNL LCD monitor, NordicNeuroLab, Bergen, Norway) and auditory stimulation was delivered to the participants through a MRConfon system (Magdeburg, Germany). A high‐resolution T1‐weighted anatomical scan was recorded in the coronal plane with 270 slices, and a voxel size of 0.86 × 0.86 × 1 mm (acquisition parameters: matrix size: 256 × 256, field of view [FOV] = 22 cm, TR = 7.3 ms, TE = 2.8 ms, flip angle = 9°, prep time = 900 ms, parallel imaging acceleration factor [PIAF] = 1.5, intensity correction: PURE). Functional T2*‐weighted echo planar images (EPI) with blood oxygenation level‐dependent contrast were acquired. A total of 264 dynamic volumes for each fMRI run were recorded with axial contiguous ascending acquisitions (voxel size: 2.3 × 2.3 × 3 mm, inter slice spacing = 0.5 mm, acquired matrix size: 96 × 96, FOV = 22 cm, number of slices: 36, TR = 2000 ms, TE = 30 ms, flip angle = 85°, PIAF = 2). To assure a steady‐state magnetization of the tissues, each scan run started with 8 s of dummy scans. Moreover, a fieldmap was acquired after the experimental task to correct the distortion of the static magnetic field during post‐processing. This required two FAST SPGR sequences with distinct Echo Time and the same space coverage as the functional EPI (TR = 50 ms, TE1 = 4.9 ms, TE2 = 7.3 ms, flip angle = 45°).

MRI data preprocessing and analyses were conducted with SPM12 (the Wellcome Trust Centre for Neuroimaging, Institute of Neurology, University College London) running on MATLAB R2016b (MathWorks, http://www.mathworks.com, MA). The following pipeline was used to preprocess the functional images: setting the origin on the anterior commissure, slice timing, computation of the voxel displacement map (VDM) (using the FieldMap2.1 toolbox, Andersson et al., 2001), spatial realignment and unwarping (using VDM previously created), normalization to the Montreal Neurological Institute (MNI) coordinate system with a voxel size of 3 × 3 × 3 mm^3^ based on the unified segmentation procedure of the co‐registered T1‐weighted anatomical image to fMRI images, and smoothing with a Gaussian kernel of 8‐mm full‐width‐at‐half‐maximum. To detect volumes with fast motion (>0.5 mm/TR), the ArtRepair toolbox was used. A general linear model was used to analyze the resulting preprocessed images at the individual subject level. The fMRI signal was modelized as condition‐specific block of 22.63 s of duration convolved with the hemodynamic response function. To remove low‐frequency noise and signal drifts, a high‐pass filter with a 1/128 Hz threshold was applied at time series from each voxel. An autoregressive function (AR(1)) was implemented to correct for temporal correlations between neighboring voxels in the whole brain.

### 2.4 Data analysis

Regarding behavioral data, accuracy (in %) and reaction times (in ms) were compared between Vowel and Stress conditions by means of paired *t*‐tests. For fMRI data, the contrast between Stress and Vowel blocks was sent to a one‐sample *t*‐test (random effect) to study the general difference of brain activity between these two conditions. The results were studied on the whole brain space with the statistical threshold family‐wise error corrected for multiple comparison at the peak level (*p*_FWE_ < 0.05) as well as at cluster level (minimal size of 11 significant contiguous voxels *p*_FDR_ < 0.05). In addition, the common activity between the two conditions within our population was studied with a conjunction over the contrasts Stress > Baseline and Vowel > Baseline.

Anatomical locations were checked with the neuromorphometrics probabilistic atlas (derived from “MICCAI 2012 Grand Challenge and Workshop on Multi‐Atlas Labeling” working with data from OASIS project http://www.oasis‐brains.org/ and labeled by http://Neuromorphometrics.com/) provided in SPM12. All the coordinates derived from these analyses are given in the MNI space, with all illustrations using the neurological convention.

## 3 RESULTS

### 3.1 Behavorial results

Results showed a higher accuracy for Vowel (96.77 %) than for Stress conditions (84.37 %; *t*(29) = 7.72, *p* < .001; Cohen's *d* = 1.41) as well as shorter reaction times for Vowel (616.44 ms) than for Stress conditions (655.94 ms; *t*(29) = −3.02, *p* = .005; Cohen's *d* = 0.55). In‐line with previous research (e.g., Dupoux et al., 1997), these findings suggest that the discrimination of stress contrasts was more difficult than the discrimination of vowel contrasts.

### 3.2 fMRI results

**FIGURE 1:**
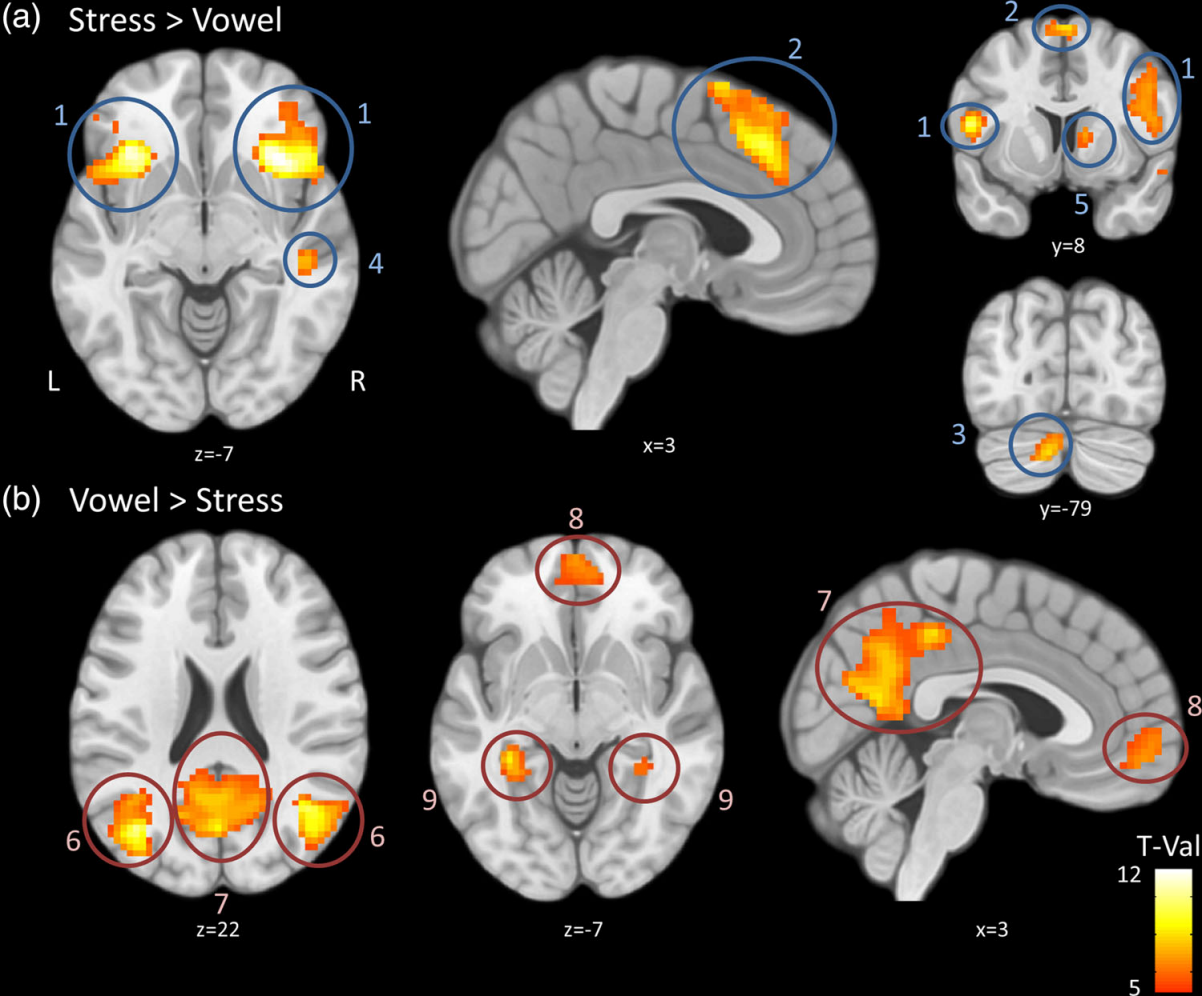
Comparison of Stress and Vowel conditions: (a) Stress > Vowel contrast with activation in (1) bilateral inferior frontal, (2) bilateral supplementary motor area, (3) right cerebellum exterior, (4) right middle/superior temporal gyrus, (5) right caudate; (b) Vowel > Stress contrast with activation in (6) bilateral middle occipital gyrus, (7) bilateral posterior cingulate gyrus, (8) bilateral medial frontal gyrus, (9) bilateral fusiform gyrus. See Table 1 for more details. Contrasts are represented with a statistic corrected at voxel level (*p*FWE < 0.05) with a minimal cluster size of 11 voxels (*p*_FDR_ < 0.05). All activation is displayed in neurological convention, and *x*, *y*, and *z* coordinates are in Montreal Neurological Institute (MNI) space. Activation color code represents the *T*‐value of the comparison.

The contrast evaluating the regions that are specifically active during stress in comparison to vowel processing primarily showed bilateral activation in the inferior frontal gyrus (IFG), the anterior insula, the frontal operculum, the orbital gyrus and temporal pole as well as in the supplementary motor area (SMA) and the anterior cingulate cortex. In addition, right hemispheric activation was found in the middle/STG and caudate and left hemispheric activation in the exterior cerebellum (see Figure 1a; Table 1A). Analysis of the inverse contrast (activity during vowel processing—activity during stress processing) evaluating the regions that are specifically active during vowel in comparison to stress processing showed bilateral activation in the middle occipital gyrus, the calcarine cortex as well as in the angular gyrus, precuneus, cuneus, posterior/middle cingulate, and in the precentral medial segment. Bilateral activation was moreover found in the frontal medial segment (see Figure 1b; Table 1B).

**TABLE 1:** Comparison of stress and vowel activation

	Coordinates (MNI)					
Anatomical location	*x*	*y*	*z*	*Z*‐max	Cluster size	Cluster coverage
***A. Stress > Vowel***						
Right anterior insula	33	23	−7	6.98	1172	BA6/9/13/44/45/46/47
Right inferior frontal gyrus	57	17	23	6.95		
Right middle frontal gyrus	51	14	35	5.93		
Left inferior frontal gyrus	−36	26	−7	6.76	478	BA13/44/45/47
Bilateral supplementary motor cortex	3	26	44	6.3	283	BA6/8/32
Left cerebellum exterior	−9	−79	−34	5.71	59	
Right middle/superior temporal gyrus	48	−28	−4	5.47	55	BA13/21/22
Right caudate	12	8	8	5.22	19	body part
***B. Vowel > Stress***						
Right middle occipital gyrus	45	−70	29	7.13	218	BA19/39
Left middle occipital gyrus	−36	−79	32	6.8	366	BA19/39/22
Bilateral posterior cingulate gyrus	−6	−34	41	6.49	1216	BA7/18/23/29/30/31
Bilateral precuneus	0	−61	17	6.13		
Left fusiform gyrus	−36	−34	−10	6.23	137	BA20/36/37/hippocampus
Right fusiform gyrus	36	−34	−16	5.62	105	BA20/36/37/hippocampus
Left inferior temporal gyrus	−54	−55	−13	5.34	12	BA20/37
Bilateral medial frontal gyrus	3	56	−10	5.28	173	BA9/10/32
Left middle frontal gyrus	−24	26	38	5.27	37	BA8
Right middle frontal gyrus	30	32	50	5.16	12	BA8
Left superior frontal gyrus	−21	11	47	5.22	20	BA6
Left middle temporal gyrus	−57	−10	−19	4.93	11	BA21
Left precuneus	−9	−58	62	4.86	14	BA7

*Note*: Contrasts was studied with a statistic corrected at voxel level (*p*FWE < 0.05) with a minimal cluster size of 11 voxels (*p*_FDR_ < 0.05).

Abbreviations: BA, Brodmann areas; MNI, Montreal Neurological Institute.

**FIGURE 2:**
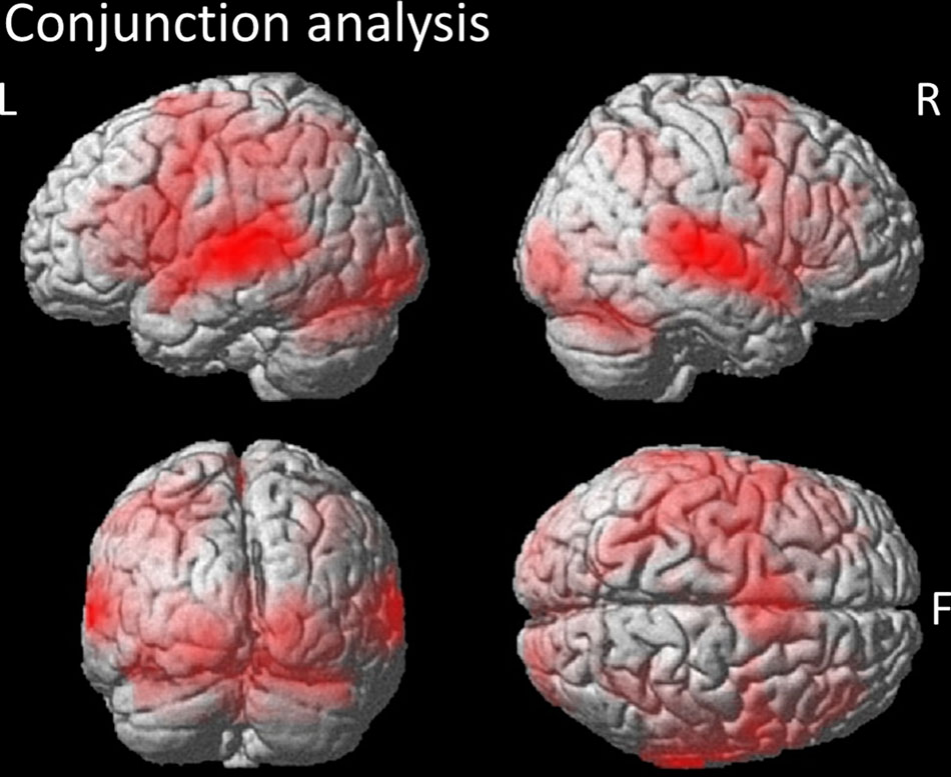
Conjunction over the contrasts Stress > Baseline and Vowel > Baseline. F, frontal pole; L, left hemisphere; R, right hemisphere. Contrasts are represented with a statistic corrected at voxel level (*p*FWE < 0.05) with a minimal cluster size of 11 voxels (*p*_FDR_ < 0.05). All activation is displayed in neurological convention.

The conjunction over the contrasts Stress > Baseline and Vowel > Baseline showed large common activation bilaterally, primarily not only in the cerebellum and the STG, but also in left and right frontal areas such as the supplementary motor cortex and subcortical areas, such as the insula and putamen (Figure 2). Please note that we refrain from including a detailed table as the conjunction is not the main question of the current research.

## 4 DISCUSSION

In the present study, we aimed at investigating the brain regions activated during stress as compared to vowel processing. To extend the knowledge from the only previous study investigating the neural basis of stress processing (Klein et al., 2011), the present research examined stress processing in an unknown language by listeners of a fixed‐stress language. To this end, French‐speaking participants without the knowledge of Spanish performed a discrimination task on Spanish word pairs differing either with respect to word stress (penultimate or final stressed word) or with respect to the final vowel while fMRI data was acquired. Given the higher difficulty of the stress versus vowel discrimination task and based on previous research suggesting stronger right‐hemisphere involvement for increasing task difficulty, we expected stronger bilateral activation of regions typically involved in word stress processing (STG, STS, pre‐/postcentral gyrus, and insula) for the stress, as compared to the vowel condition. Moreover, we also expected frontal areas typically associated with domain general cognitive control to be more strongly involved in the Stress > Vowel processing.

Among the areas typically associated with word stress processing, we indeed found differences in activation in the bilateral SMA, the insula as well as the middle/STG. The bilateral SMA activation corroborates results from a previous study investigating the processing of word prosody (Kandylaki et al., 2017). Bilateral SMA activation, together with STG and IFG activations, seems to increase with greater phonological working memory load (Perrachione et al., 2017). Moreover, the SMA seems to play a role not only in speech motor control as an initiation and timing interface, but also in verbal working memory and inner silently articulated speech (Hertrich et al., 2016). Bilateral SMA activation has been linked to the processing of speech rhythm (Geiser et al., 2008) as well as temporal sequencing of complex acoustic nonspeech information (Schwartze et al., 2012).

The higher bilateral activation of the anterior insula could be associated with more demanding sound detection and auditory temporal processing (Uddin et al., 2017 for a review). A meta‐analysis by Sallet et al. (2012) showed that the insula is part of a network responsible for difficult speech processing, not only during speech production but also during comprehension of distorted speech, suggesting that this area is recruited under difficult challenging listening conditions. Another result that might be reflective of the higher difficulty and therefore the cognitively more demanding process of discriminating stress contrasts is the finding of stronger activation in the right middle/STG. Although several previous studies on metrical stress/phonological processing revealed left hemispheric dominance (Aleman et al., 2005), in most cases, bilateral activation (Domahs et al., 2013; Heisterueber et al., 2014; Klein et al., 2011) was reported, especially with increased task difficulty (Domahs et al., 2013; Klein et al., 2011). Moreover, the result is in‐line with other studies claiming possible right hemispheric dominance in prosodic processing such as complex sound analysis (Gandour et al., 2004).

As already mentioned, based on the assumption that the stress condition is more difficult than the vowel condition, we also expected stronger involvement of areas associated with cognitive control for the Stress > Vowel condition. In the present study, we found such stronger activation in the bilateral IFG for Stress > Vowel processing, slightly more pronounced in the right hemisphere, a result that was similar in Klein et al. (2011). Bilateral activation in the IFG has previously been linked to regulation behavior in demanding tasks requiring to resolve competition among different characteristics of linguistic stimuli (Novick et al., 2010 for a review). Increased left IFG recruitment under high versus low demands in different language tasks has been observed in several previous studies using picture naming tasks, lexical decision tasks or phonological and semantic judgment tasks (Grindrod et al., 2008; Kan & Thompson‐Schill, 2004; Schnur et al., 2009; Snyder et al., 2007). In‐line with this idea of a “control function” of Broca's area, a recent publication by Fedorenko & Blank (2020)bib15 postulates that the left IFG is structurally and functionally heterogenous and can be divided into two subregions, one being part of the domain‐specific “language network” and the other one belonging to the domain‐general “multiple‐demand network”. According to the authors, the “language network” seems to be associated with “high‐level” language processing, such as lexical and syntactic and/or semantic processings (Fedorenko et al., 2010, 2012), whereas the multiple‐demand network seems more related to domain‐general attentional and executive processings (e.g., Cole & Schneider, 2007; Fedorenko & Blank, 2020 for a review). Apart from the involvement of the IFG in such domain‐general executive processing, right‐hemispheric IFG activation has previously been associated with language domain‐specific processing, such as the processing of pitch (Hsieh et al., 2001), prosody (Sammler et al., 2015), and accent patterns (Geiser et al., 2008).

The differential activation of the left cerebellum and the caudate nucleus for Stress > Vowel processing seems less straightforward. However, the cerebellum has been not only shown to be associated with a wide range of cognitive functions, including sensorimotor, language, and working memory (Ashida et al., 2019), but also more specifically with predictive language processing (Moberget et al., 2014) as well as prosody processing (Hernández et al., 2019). Regarding the different activation in the caudate nucleus, clinical studies with patients showing language and speech impairment suggest that the caudate nucleus participates in the control of language and speech processing (Grönholm et al., 2016), and that it is also a part of the network involved in domain‐general language control in bilinguals (Nair et al., 2021). This latter point is again in‐line with the assumption that the stress contrast is cognitively more demanding than the vowel contrast.

The contrast between Vowel > Stress conditions revealed differences in activation in a network primarily comprising the bilateral posterior cingulate and the adjacent precuneus, the bilateral fusiform gyrus and hippocampus, the bilateral middle occipital gyrus as well as the bilateral medial frontal gyrus. The largest cluster that showed stronger activation for Vowel > Stress conditions was found in the bilateral posterior cingulate and the precuneus as well as regions in the temporal cortex including hippocampal and parahippocampal regions. Interestingly, these are all regions typically associated with the default mode network, which is known for increasing its activity when tasks require less attention (Raichle, 2015 for a review).

In the conjunction analyses, we were able to replicate the results of Klein et al. (2011), this time with a group of participants without expertise in stress processing, showing large overlapping regions for stress and vowel processing in a network of bilateral frontotemporal and cerebellar regions. In the study by Klein et al. (2011), who investigated stress processing in German‐speaking participants listening to pseudowords, there was no possible lexical access. In the present study, given that several of the Spanish words were cognates of French words, we cannot exclude a lexical access of these cognates in the L1‐French participants of the present study. Nevertheless, the replication of the results revealing large overlapping regions for stress and vowel processing in bilateral frontotemporal and cerebellar regions seems interesting as it reflects the activation pattern during auditory processing of prosodic patterns when no or little lexical processing is involved.

Taken together, the results show that when processing stress in a foreign free‐stress language, native speakers of a fixed‐stress language activate not only a domain‐specific “language network” during prosody processing, but also a domain general anterior network including regions related to cognitive control and working memory. On the other hand, they show a decrease in regions related to the default mode network, most likely reflecting the higher demand during stress processing. This higher level of attention and cognitive control for stress than vowel processing was further supported by the behavioral results showing lower accuracy and longer reaction times for discriminating stress contrasts than vowel contrasts.

## CONFLICTS OF INTEREST

No conflicts of interest.

### PEER REVIEW

The peer review history for this article is available at: https://publons.com/publon/10.1002/brb3.2854.

## Data Availability

The data that support the findings of this study are openly available in Zenodo at https://doi.org/10.5281/zenodo.7031880.

## NOTE

1 The underlined syllable in these examples, and in the rest of the paper, corresponds to the stressed syllable.
